# Genetic Origin and Introgression Pattern of Pingliang Red Cattle Revealed Using Genome-Wide SNP Analyses

**DOI:** 10.3390/genes14122198

**Published:** 2023-12-11

**Authors:** Yuanqing Wang, Jun Ma, Jing Wang, Lupei Zhang, Junwei Hu, Minghao Ma, Lingyang Xu, Yan Chen, Bo Zhu, Zezhao Wang, Huijiang Gao, Junya Li, Xue Gao

**Affiliations:** 1 Laboratory of Molecular Biology and Bovine Breeding, Institute of Animal Science, Chinese Academy of Agricultural Sciences, Beijing 100193, China; 82101225454@caas.cn (Y.W.);; 2 Academy of Pingliang Red Cattle, 492 South Ring Road, Kongtong District, Pingliang 744000, China

**Keywords:** Pingliang red cattle, population genetics, genetic origin, introgression pattern

## Abstract

The Pingliang red cattle, an outstanding indigenous resource in China, possesses an exceptional breeding value attributed to its tender meat and superior marbling quality. Currently, research efforts have predominantly concentrated on exploring its maternal origin and conducting conventional phenotypic studies. However, there remains a lack of comprehensive understanding regarding its genetic basis. To address this gap, we conducted a thorough whole-genome analysis to investigate the population structure, phylogenetic relationships, and gene flows of this breed using genomic SNP chip data from 17 bovine breeds. The results demonstrate that Pingliang red cattle have evolved distinct genetic characteristics unique to this breed, clearly distinguishing it from other breeds. Based on the analysis of the population structure and phylogenetic tree, it can be classified as a hybrid lineage between *Bos taurus* and *Bos indicus*. Furthermore, Pingliang red cattle display a more prominent *B. taurus* pedigree in comparison with Jinnan, Qinchuan, Zaosheng, Nanyang, and Luxi cattle. Moreover, this study also revealed closer genetic proximity within the Chinese indigenous cattle breed, particularly Qinchuan cattle, which shares the longest identical by descent (IBD) fragment with Pingliang red cattle. Gene introgression analysis shows that Pingliang red cattle have undergone gene exchange with South Devon and Red Angus cattle from Europe. Admixture analysis revealed that the proportions of East Asian taurine and Chinese indicine in the ancestry of Pingliang red cattle are approximately 52.44% and 21.00%, respectively, while Eurasian taurine, European taurine, and Indian indicine account for approximately 17.55%, 7.27%, and 1.74%. Our findings unveil distinct genetic characteristics in Pingliang red cattle and attribute their origin to *B. taurus* and *B. indicus* ancestry, as well as contributions from Qinchuan cattle, South Devon, and Red Angus.

## 1. Introduction

China is home to approximately 10 million indigenous cattle, which are classified into 56 distinct breeds raised in diverse agroecological environments. The diversity and unique characteristics of these cattle have also been influenced by a complex historical culture. Pingliang red cattle, a native species from Gansu province, has been profoundly affected by the Hexi Corridor and has served as a crucial link between Central China and the rest of Eurasia for several millennia. This corridor enabled trans-Eurasian cultural exchange throughout prehistoric history. Consequently, Pingliang red cattle have developed multi-paternal lines from Limousine, South Devon, Angus, and Qinchuan cattle, among others [1]. Although extensive gene mapping and diversity studies have been reported on this breed, the population structure and evolutionary history of Pingliang red cattle remain poorly investigated.

Previous studies have predominantly focused on the matrilineal origin of Pingliang red cattle. Zhao et al. (2013) sequenced the mtDNA D-loop HVS region of 88 Pingliang native cattle to determine their haplotypes and analyze their phylogenetic relationships with 22 other Chinese local breeds. The results demonstrated that ninety-five haplotypes were identified and distributed into two major phylogenetic groups representing the distinct mtDNA genomes of *B. taurus* and *B. indicus*, respectively. These findings indicate that Pingliang cattle are derived from two maternal genetic backgrounds [2]. Liu et al. (2014) conducted an investigation on the genetic diversity of Zaosheng cattle and analyzed six groups of Zaosheng cattle using mtDNA D-loop analysis. A total of 131 variable sites and 103 haplotypes were identified. The findings revealed that the Pingliang population of Zaosheng cattle (known as Pingliang red cattle) originated from Qingyang and gradually developed distinct maternal lineages [3]. In 2018, Cai et al. conducted an assessment of the maternal genetic and phylogenetic characteristics of domesticated cattle by analyzing the hypervariable segment I (HVS-I) of mitochondrial DNA in 698 native cattle from eight areas in northwestern China. The findings revealed that the native cattle in this region were derived from two distinct maternal ancestors, namely *B. taurus* and *B. indicus*, which migrated to central China from the northern and southern areas, respectively. It is worth noting that while *B. taurus* remained at the periphery of the region, a population expansion event occurred in the Longdong region of Gansu Province, resulting in four relatively independent evolutionary branches. Following this expansion event, there was the subsequent migration of *B. indicus* from southern to northern China [4].

Population genomics, the study of genome sequence variation within and between closely related species [5], provides a comprehensive perspective on selection and genetic drift in evolutionary processes [6]. Additionally, population genomics offers invaluable insights into the biological traits of organisms that are specifically influenced by adaptive evolution. The matrilineal and patrilineal origins of these species were previously determined by examining genetic variation in the mitochondrial and Y-chromosome genomes [7]. However, recent advancements in high-throughput and cost-effective genotyping techniques have enabled a comprehensive genome-wide assessment of the genetic structure and relationships among cattle populations [8].

Gibbs et al. (2009) genotyped 37,470 single-nucleotide polymorphisms (SNPs) in 497 cattle from 19 geographically and biologically diverse breeds worldwide. The findings unveiled a recent and rapid decline in the effective population size of cattle, indicating the discernible impact of domestication and artificial selection on the detectable signatures of selection within the bovine genome [9]. Gautier et al. (2010) conducted a comprehensive assessment of cattle genetic diversity by genotyping 1121 individuals from 47 populations for 44,706 autosomal SNPs. The results indicated that domestic cattle populations can be categorized into the following three primary groups: African taurine, European taurine, and zebus. Furthermore, the analysis of spatial patterns of genetic diversity corresponds to the two main migration routes leading to France [10]. The population structure of 134 domesticated bovid breeds was assessed using genotypes from 43,043 autosomal single nucleotide polymorphism markers obtained from 1543 animals in another study. The results presented compelling evidence supporting the classification of domestic cattle populations into the following three primary groups: Asian indicine, Eurasian taurine, and African taurine. Additionally, an analysis of the population history and structure of Iranian cattle revealed no strong introgression between Iranian cattle and other global breeds [11,12]. Gao et al. (2017) discovered a discontinuous distribution of taurine and indicine cattle ancestries, characterized by less than 10% indicine cattle in the northern regions and exceeding 90% in the southern and southwestern parts of China by 50 K SNP genotyping [13]. The utilization of model-based clustering and f4-statistics reveals the occurrence of introgression from both banteng and gayal into cattle populations in southern China, while the sporadic presence of yak’s genetic influence among cattle inhabiting or neighboring Tibetan regions supports earlier findings derived from mitochondrial DNA analysis. Xu et al. (2019) genotyped a total of 179 samples from eight cattle breeds, namely Yanhuang, Menggu, Caidamu, Liangshan cattle, Pingwu, Zhaotong, Wenshan and Nandan cattle, using the genome-wide bovine genotyping array kit (BovineHD SNPs array, Illumina, San Diego, CA, USA) containing 777,962 SNPs. The findings revealed a significant correlation between the genetic structures of these populations and their respective geographic locations, thereby enabling the identification of a distinct set of group-specific and breed-specific candidate genes [14].

The population structure and selection signatures of global and regional cattle populations have been extensively investigated in previous studies. The current research on the Pingliang red cattle primarily focuses on maternal genetic origin and conventional phenotypic genetic studies. However, there is a lack of comprehensive understanding regarding the genetic basis of the Pingliang red cattle population’s structure and its phylogenetic relationships with other global cattle breeds. In this study, we utilized genomic SNP chip data to thoroughly investigate the genetic structure of Pingliang red cattle, evaluating genetic diversity and population structure and elucidating phylogenetic relationships with other cattle species.

## 2. Materials and Methods

### 2.1. Samples Collection and Genotyping

The blood samples were collected from 122 Pingliang red cattle in Gansu Province, China. Additionally, the frozen semen samples of the beef cattle breeds used in the hybrid improvement of Pingliang red cattle, including Qinchuan cattle (QIC, n = 16), Red Angus (RAG, n = 7), South Devon (SDE, n = 8), and Limousin (LMS, n = 9) were obtained. The Qinchuan cattle were genotyped using the Illumina BovineHD SNPs array, while the remaining samples were performed using the GGP Bovine 100 K SNP chip (including a total of 95,256 SNPs).

Additionally, 50 K genotyping data of related cattle breeds, such as *B. indicus* and *B. taurus*, previously published in the literature, were retrieved from publicly available databases. These include Qinchuan cattle (QIC, n = 15), Yanbian cattle (YAB, n = 26), Mongolian cattle (MGX, n = 31), Jinnan cattle (JIN, n = 14), Luxi cattle (LUX, n = 11), Nanyang cattle (NAY, n = 23), Limousin (LMS, n = 21), Red Angus (RAG, n = 19), Hanwoo (HAN, n = 8), Gir (GIR, n = 24), Nellore (NEL, n = 21), and Banteng (BLI, n = 20) from the WIDDE database [10,13,15,16].

(http://widde.toulouse.inra.fr/widde/widde/main.do;jsessionid=114993EC02C5CB17D1FA786DD4F2337A?module=cattle# (accessed on 12 May 2022)). The genotyping data of Jinnan cattle (JIN, n = 14), Jian cattle (JIA, n = 18), and Leiqiong cattle (LEQ, n = 12) comprising 770 K markers were acquired from the NCBI GEO database. Furthermore, the genotyping data for Zaosheng cattle (ZAS, n = 10) were included in this study as they had previously undergone genotyping using the 770 K chips (Table 1).

### 2.2. Quality Control

The Plink software (v1.9, https://www.cog-genomics.org/plink/ (accessed on 6 May 2022)) was utilized to implement quality control (QC) by excluding individuals and filtering out SNPs [17,18,19,20]. The QC procedure involved the exclusion of individuals with more than 10% missing genotypes and SNPs with call rates lower than 0.95, minor allele frequencies (MAF) below 0.05, or a Hardy–Weinberg equilibrium (HWE) less than 10^−6^. Additionally, sex chromosome loci and repeat SNP loci were removed, while closely related individuals (PIHAT values above 0.25) were also excluded [21]. Ultimately, a total of 18,657 autosomal SNPs obtained from 443 individuals and representing 17 distinct cattle populations were used for analysis.

**Table 1:** Sample information of 17 cattle populations.

Breed	Abbr.	Chip Type	Geographical Region	Number of Pre-QC(N)	Number of Post-QC(N)	Source
Pingliang red	PLC	100 K	Gansu, China	122	122	this study
Zaosheng	ZAS	770 K	Gansu, China	10	10	this study
Qinchuan	QIC	50 K	Shaanxi, China	15	15	Gao et al. [13]
		770 K		16	16	this study
Jinnan	JIN	50 K	Shanxi, China	14	13	Gao et al. [13]
		770 K		14	14	Zhang et al. [22]
Mongolian	MGX	50 K	Inner Mongolia Autonomous Region, China	31	30	Gao et al. [13]
Yanbian	YAB	50 K	Jilin, China	26	26	Gao et al. [13]
Luxi	LUX	50 K	Shandong, China	11	11	Gao et al. [13]
Nanyang	NAY	50 K	Henan, China	23	23	Gao et al. [13]
Jian	JIA	50 K	Jiangxi, China	18	16	Zhang et al. [22]
Leiqiong	LEQ	50 K	Guangdong, China	12	12	Zhang et al. [22]
Limousin	LMS	50 K	France	21	21	this study
		100 K		9	9	Gautier et al. [10]
South Devon	SDE	100 K	England	8	7	this study
Red Angus	RAG	50 K	England	19	19	this study
		100 K		7	7	Gao et al. [13]
Hanwoo	HAN	50 K	Korea	8	8	Decker et al. [15]
Gir	GIR	50 K	India	24	24	http://www.illumina.com/applications/agriculture.ilmn (accessed on 12 May 2022)
Nellore	NEL	50 K	Brazilian	21	21	Matukumalli et al. [16]
Banteng	BLI	50 K	Indonesia	20	19	Gao et al. [13]
Total				449	443	

### 2.3. Principal Component and Population Structure Analysis

Principal component analysis (PCA) was carried out incorporating all available SNP information using the Plink v1.9. Genome-wide identity-by-state (IBS) pairwise distances were estimated to cluster the samples using Plink v1.9 (-mds -plot 4) [23]. Subsequently, the eigenvectors were derived from the inferred covariance matrix and utilized to generate a PCA biplot with ggplot2 (R Packages) [24]. The population structure was carried out using the software admixture (v1.3.0, http://dalexander.github.io/admixture/index.html (accessed on 15 May 2022)), which employs a model-based estimation of individual ancestry across a range of user-defined prior values of K. To account for the potential population structure within the breeds, the values of K ranged from 2 to 9. The results were visualized using the R package pophelper.

### 2.4. Phylogenetic Tree Analysis

The genetic distance between the pairwise combination of individuals was estimated using Plink v1.9 with the “-distance-matrix” option, and a neighbor-joining phylogenetic tree was constructed using PHYLIP v3.69 [8]. The resulting phylogenetic tree was visualized with Figtree 1.3.1. In the analysis, Banteng (*B. javanicus*) was used as an outgroup, as previously described [25,26]. To further investigate the population-level phylogenetic relationships between Pingliang red cattle and other groups, a population-based maximum-likelihood (ML) phylogenetic tree comprising 17 cattle populations was constructed using Treemix with parameters set to “-k 1000 -global” [27].

### 2.5. Identity by Descent Analysis

The genome-wide distances of identity by descent were estimated for the clustered samples using IBDLD v3.37. The parameters were set as “-Plinkbf_int file -method GIBDLD -ploci10 -step 0 -hiddenstates 3 -nthrcads 30 -segment -length 10 min 0.8”. The calculation of normalized IBD (nIBD) between Pingliang red cattle and each population was performed as follows: nIBD = cIBD/tIBD, where cIBD represents the count of all haplotypes shared by Pingliang red cattle and each population, and tIBD denotes the total pairwise comparisons between Pingliang red cattle and each population.

### 2.6. TreeMix Analysis

The Treemix v. 1.13 software was utilized to infer historical relationships, based on splits and migrations (mixtures), between Pingliang red cattle and other populations using genome-wide polymorphisms [28]. We rooted the graphs with Banteng as the outgroup, used blocks of 1000 SNPs, and applied the “-se” option to calculate the standard errors of migration proportions. TreeMix was iteratively executed for values of the migration parameter “-m” ranging from 1 to 20. The f index, which represents the proportion of variance in the sample covariance matrix compared to the model covariance matrix, was employed to determine the optimal model for gene migration pathways [29].

## 3. Results

### 3.1. Principal Component and Population Structure Analysis

**Figure 1:**
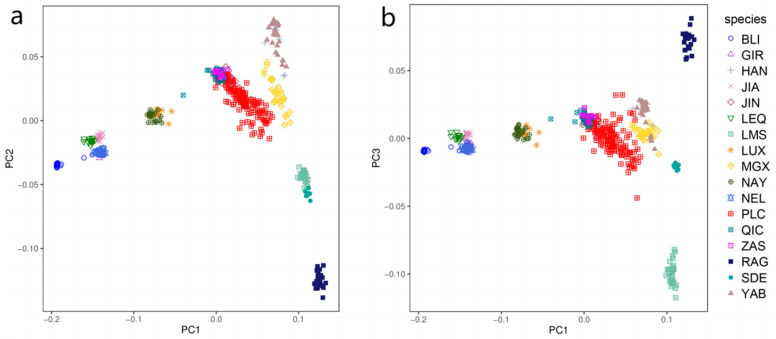
Principal component analysis of 443 individuals from 17 cattle populations: (**a**) PC1 and PC2 results of principal component analysis; (**b**) PC1 against PC3.

We performed PCA on a total of 433 individuals representing 17 cattle populations. The first two principal components (PCs) are illustrated in Figure 1a. The PC1 clearly demonstrates the distinct positioning of *B. taurus* and *B. indicus* at opposite ends, providing an explanation for their genetic divergence. Due to their close geographical proximity, the Pingliang red cattle exhibit significant genetic relatedness to Qinchuan, Zaosheng, and Jinnan cattle [30]. Conversely, the results of PC2 reveal disparities between eastern Asia and European taurine cattle, as well as variations between Chinese indicine cattle and those from Indian indicine. Figure 1b illustrates that PC1 and PC3 can effectively discriminate between British taurine breeds (Red Angus and South Devon) and the France taurine breed (Limousin).

**Figure 2:**
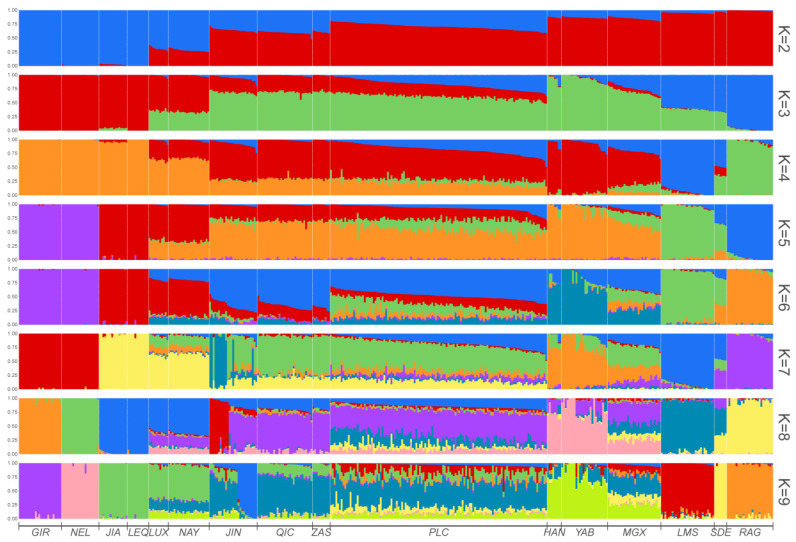
Analysis of population ancestry composition when K = 2–9.

To determine the level of admixture, we conducted an admixture analysis by varying the number of clusters (K) from 2 to 9 (Figure 2). When K = 2, the clustering pattern revealed a remarkable division of *B. taurus* and *B. indicus*. Pingliang red cattle exhibited a mixed ancestral composition of both *B. taurus* and *B. indicus*, with a higher proportion of taurine cattle ancestral components compared to local Chinese cattle breeds such as Luxi, Nanyang, Qinchuan and Zaosheng cattle. When K = 3, the results were consistent with PC2 analysis and demonstrated a clear differentiation between eastern Asia and European taurine cattle populations. At this value of K, Pingliang red cattle displayed a certain proportion of taurine ancestral components from European cattle that were not observed in the Luxi, Nanyang, Qinchuan and Zaosheng cattle. When K = 4, Pingliang red cattle showed greater heterogeneity with additional ancestral components from two European cattle populations that were absent in Qinchuan and Zaosheng cattle. When K = 5, Jian and Leiqiong cattle in southern China did not exhibit any ancestral component from *B. indicus*, which is consistent with previous studies [31]. Furthermore, based on geographic location, the different cattle populations in our study could be classified into the following five ancestral components: European taurine (Red Angus), Eurasian taurine (Limousin), East Asian taurine (Han and Yanbian cattle), Chinese indicine (Jian and Lei Qiong cattle) and Indian indicine (Gir and Nellore). Our findings also indicate that approximately 52.44% of the ancestry of Pingliang red cattle can be attributed to the East Asian taurine lineage, while 21.00% can be traced back to the Chinese indicine lineage. Additionally, around 17.55%, 7.27%, and 1.74% of their genetic makeup can be linked to Eurasian taurine, European taurine, and Indian indicine lineages, respectively. Finally, with K = 9, it was discovered that apart from sharing common ancestral components with Qinchuan cattle, Pingliang red cattle possess a certain percentage of shared ancestral components with South Devon, Red Angus, and Limousin, respectively. In summary, Pingliang red cattle have developed their own unique genetic structure in terms of group composition characteristics and exhibit evident heterogeneity compared to other cattle breeds.

### 3.2. Phylogenetic Analysis of Pingliang Red Cattle with Other Breeds

**Figure 3:**
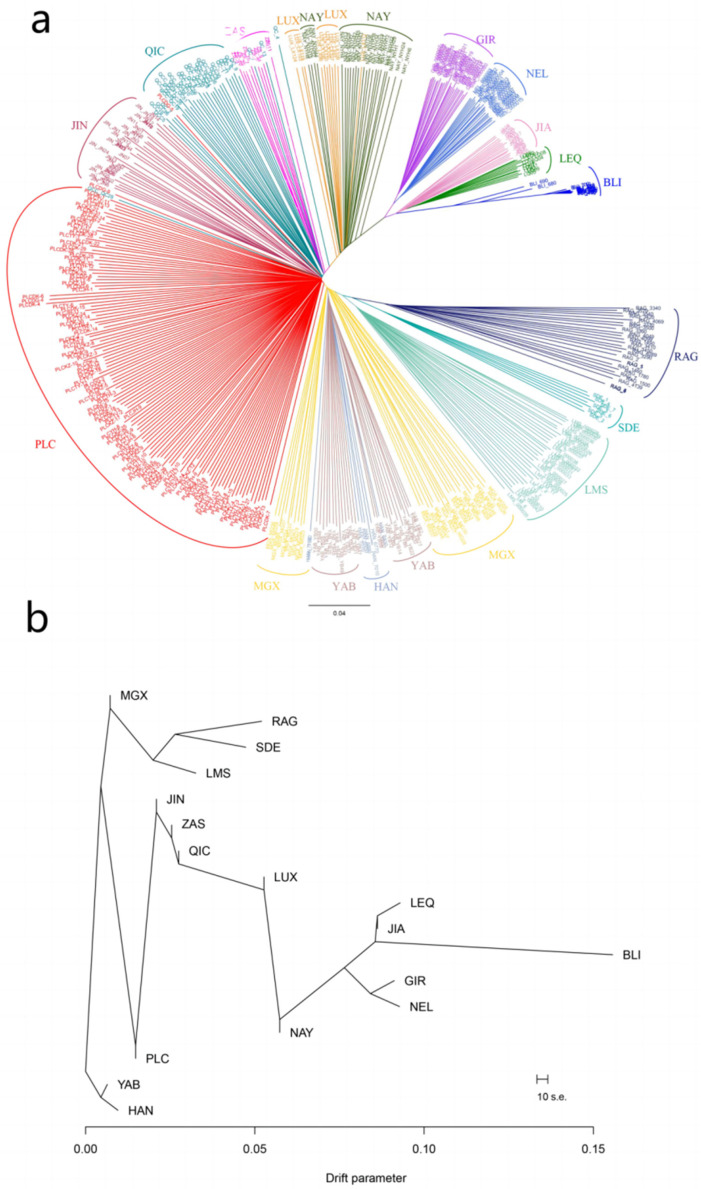
Population genetic relationship analyses for 443 individuals representing 17 different bovine breeds: (**a**) Phylogenetic tree constructed using the neighbor-joining method; (**b**) Maximum-likelihood phylogenetic trees.

Additionally, in order to investigate the genetic relationships among different individuals, a neighbor-joining (NJ) tree was constructed using Banteng cattle as the outgroup (Figure 3a). It was observed that animals from the same populations tended to cluster together, with *B. taurus* and *B. indicus* positioned at opposite ends of the NJ tree. Notably, Pingliang red cattle occupied an intermediary position between *B. taurus* and *B. indicus*, along with other Chinese cattle populations such as Nanyang, Luxi, Qinchuan, and Jinnan cattle. These findings indicate that Pingliang red cattle have mixed ancestral components derived from both *B. taurus* and *B. indicus*. Consistent with the results obtained from principal component analysis (PCA), Chinese cattle populations exhibiting a similar composition of taurine–indicine mixtures were found to be relatively close in their relation to Pingliang red cattle; examples include Qinchuan, Zaosheng, and Jinnan cattle. However, in comparison to the results obtained using PCA analysis, individuals within the Pingliang red cattle populations exhibited distinct clustering patterns that clearly distinguished them from other breeds. To further explore the possible interbreeding events across populations, we constructed a maximum-likelihood (ML) tree incorporating the admixture using TreeMix (Figure 3b). Consistent with the NJ tree results, the distributional characteristics of different cattle populations aligned with their geographic location. Noteworthy, our genetic analysis revealed a closer relationship between Pingliang red cattle and taurine cattle originating from Northeast Asia and Europe compared to Jinnan, Qinchuan, and Zaosheng cattle. This can be attributed to the historical introduction of taurine cattle from Europe during the breeding process aimed at improving Pingliang red cattle.

### 3.3. Introgression Pattern of the Pingliang Red Cattle

**Figure 4:**
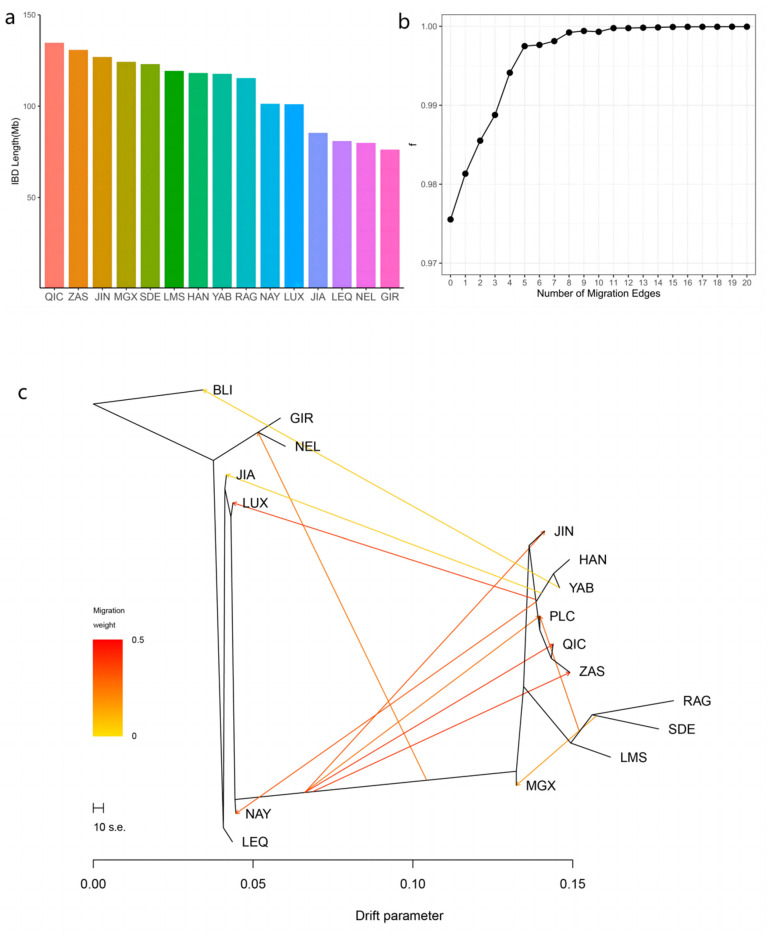
Introgression pattern of Pingliang red cattle: (**a**) the length of the shared haplotype lineage (IBD) fragment between Pingliang red cattle and other breeds; (**b**) f-value analysis of the optimal migration model among 17 cattle populations; (**c**) optimal population migration model inferred using Treemix software v. 1.13 (m = 11).

The results of IBD analysis showed a close genetic relationship between Qinchuan cattle and other Chinese bovine breeds. In particular, Pingliang red cattle exhibited significantly higher levels of shared IBD with Qinchuan cattle compared to other populations, with an IBD segment length of 134.69 Mb. Conversely, Gir displayed the smallest shared IBD segment with Pingliang red cattle, measuring 76.23 Mb (Figure 4a).

**Figure A1:**
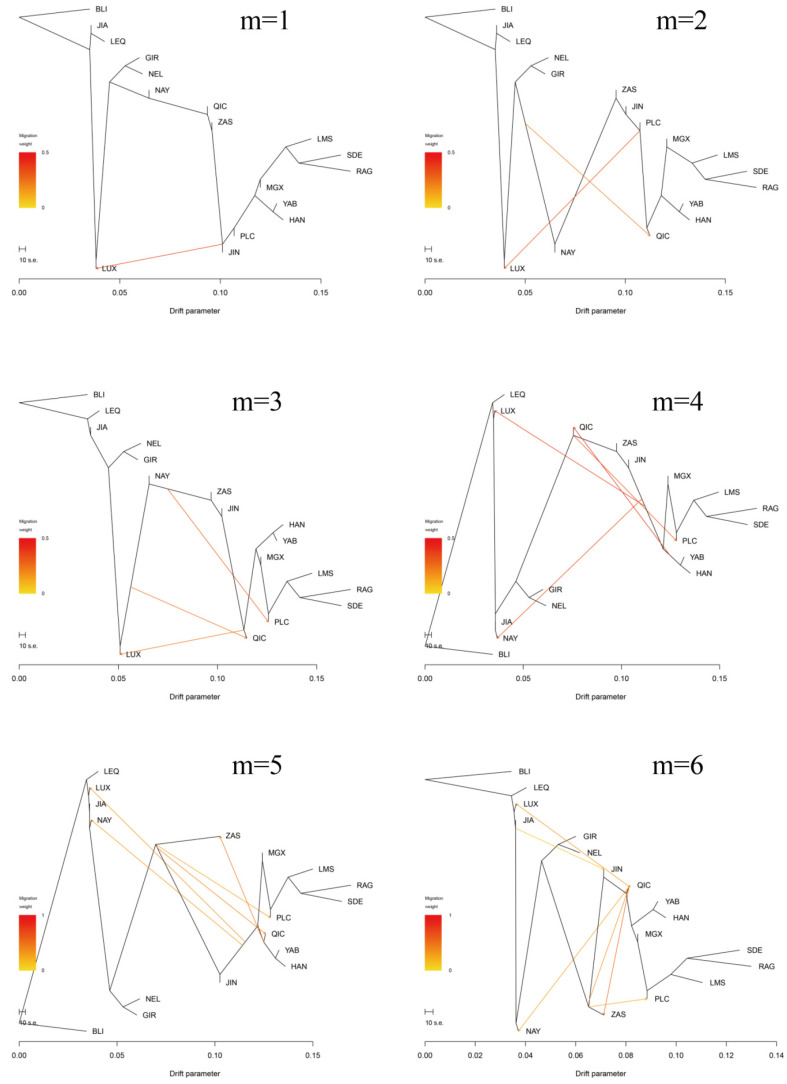

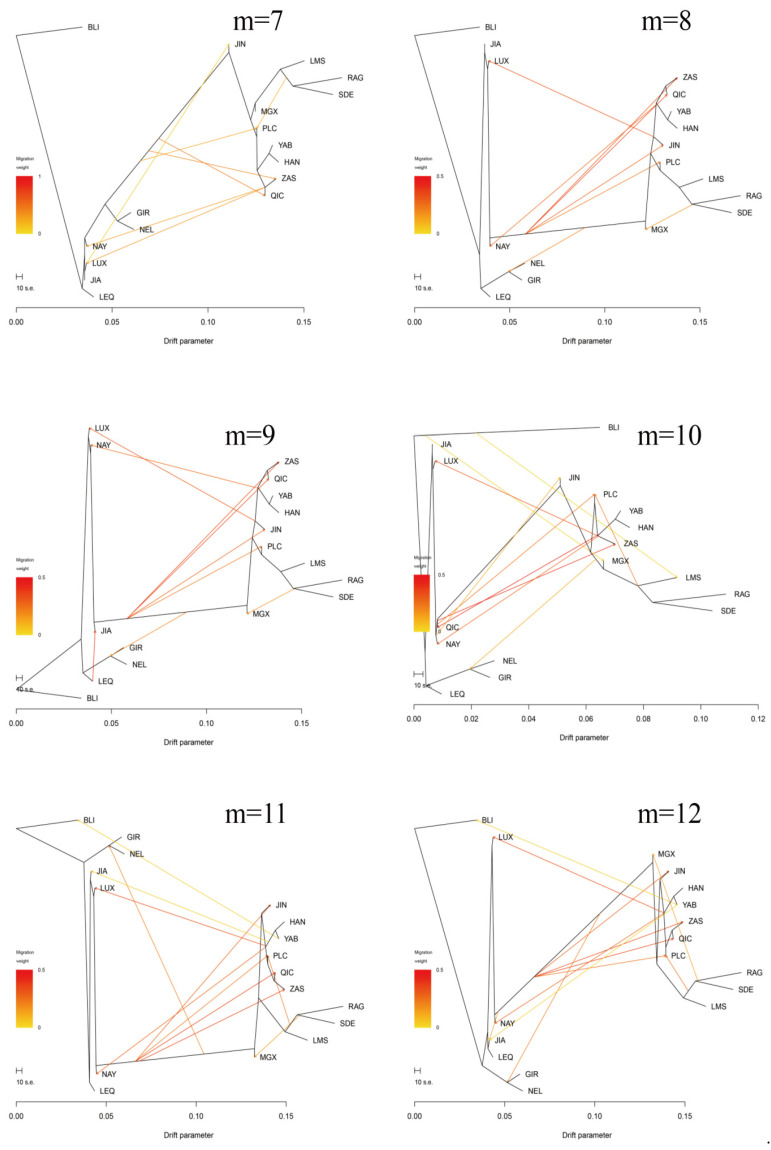

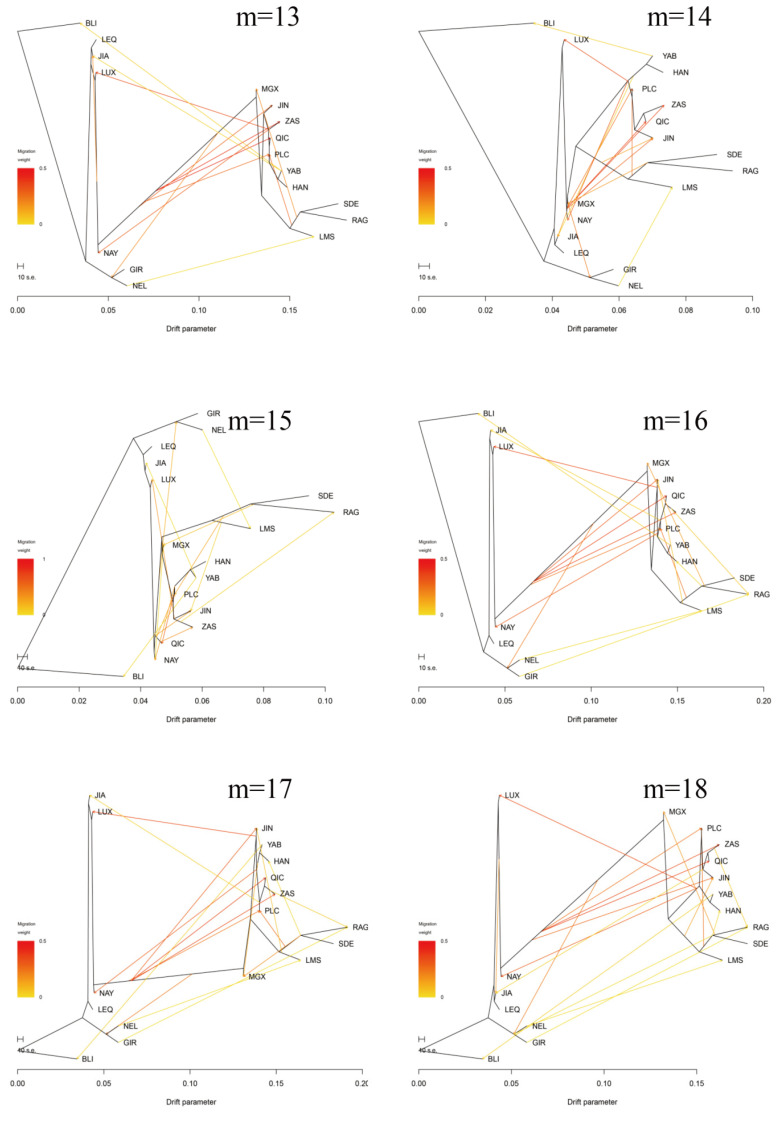

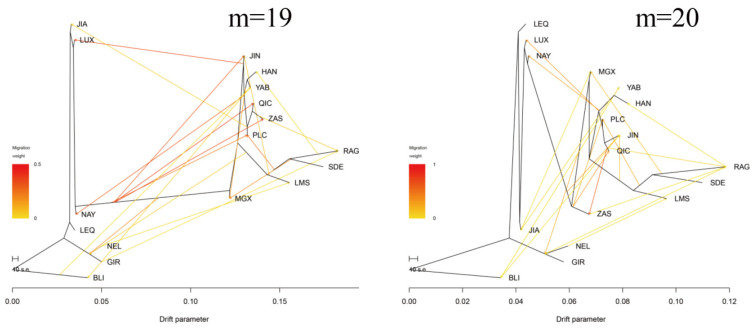
Treemix analysis of 17 cattle populations (m = 1–20).

Furthermore, the Treemix gene was employed to analyze gene flow between Pingliang red cattle and other breeds (Figure 4b,c, Appendix A Figure A1). Consistent with the results of admixture analysis, we also observed evidence of gene flow from the *B. indicus* to Pingliang red cattle when considering the migration edges of 3, 4, 5, and 6. Moreover, an examination of migration edge 7 revealed that South Devon and Red Angus exhibited a gene flow towards Pingliang red cattle, in addition to the previously mentioned gene flow from the *B. indicus* to Pingliang red cattle. The f-value curve gradually smoothed out after reaching migration edge 11; therefore, we considered this to be the optimal mode for depicting gene flow routes. Within this optimal gene flow routes, from Yanbian cattle to Banteng (0.041), East Asian to Jian cattle (0.043), and South Devon to Mongolian cattle (0.176) were excluded due to their migration weight values being less than 0.2, which indicates a significant level of presence in the gene flow. In addition, we identified gene flow from *B. indicus* toward Jinnan, Qinchuan, and Zaosheng cattle, as well as gene flow from *B. taurus* toward Luxi, Nanyang cattle, and Nellore.

## 4. Discussion

To investigate the distinct genetic structure of Pingliang red cattle and its genetic relationship with other breeds, we conducted a comprehensive analysis. Firstly, admixture, neighbor-joining, and maximum-likelihood methods were employed to examine the phylogenetic relationships among different populations. Additionally, identical by descent and Treemix were utilized to infer the common genetic ancestry and gene flow among these populations. In this study, we integrated gene chip data from Pingliang red cattle and other cattle populations, including their lineage groups [22,32,33]. After rigorous quality control procedures, a total of 443 individuals with 18,657 SNPs were involved in the subsequent analyses. The results obtained from the PCA and phylogenetic NJ tree analysis confirmed that the distribution of all individuals was consistent with their geographic location. Notably, individuals from Pingliang red cattle were able to visibly congregate together, clearly distinguishing them from other cattle populations. Furthermore, admixture analysis revealed that when K = 2 (the number of ancestral components), Pingliang red cattle displayed similar mixed ancestry patterns to Chinese cattle such as Jinnan, Nanyang, Qinchuan, and Zaosheng cattle, which are known to be a mixture of *B. taurus* and *B. indicus*; however, Pingliang red cattle contained a higher percentage of taurine ancestral components compared to these local breeds [34,35,36]. For K greater than or equal to three (indicating more refined population subdivisions), Pingliang red cattle demonstrated its own unique population genetic structure composition characterized by a significant proportion of the European cattle population ancestry in contrast to the geographically proximate Qinchuan, Zaosheng, and Jinnan populations. The distinctive population genetic structure composition became even more evident with increasing K values.

Combining the results of the NJ and ML phylogenetic tree analyses, it is evident that Pingliang red cattle have a genetic composition that is a mixture of *B. taurus* and *B. indicus* due to the historical introduction of taurine cattle from Europe for breed improvement during breeding processes. As a result, Pingliang red cattle possess a significant proportion of European ancestry components, establishing closer genetic relatedness with taurine cattle from Northeast Asia and European regions compared to Jinnan, Qinchuan, and Zaosheng cattle. Additionally, our admixture analysis with K = 5 revealed the following five ancestral components: European taurine (Red Angus), Eurasian taurine (Limousin cattle), East Asian taurine (Hanwoo and Yanbian cattle), Chinese indicine (Jian and Lei qiong cattle), and Indian indicine (Gir and Nellore). These findings are consistent with Chen’s classification of ancestry in worldwide cattle populations using whole genome resequencing data [31]. Currently, the proportions of East Asian taurine and Chinese indicine in the ancestry of Pingliang red cattle are approximately 52.44% and 21.00%, respectively, while Eurasian taurine, European taurine, and Indian indicine account for approximately 17.55%, 7.27%, and1.74%. Furthermore, our analysis using Treemix indicates gene flow between Pingliang red cattle with South Devon and Red Angus breeds. This finding aligns with both the genetic structure analysis conducted through admixture as well as the breeding history documentation regarding Pingliang red cattle [37].

To further investigate the genetic distinctions between Pingliang red cattle and other cattle populations, we conducted additional analysis on the shared identity by descent (IBD) fragments between Pingliang red cattle and other breeds. The results of the shared IBD fragment analysis, along with the findings from principal component analysis (PCA), demonstrated that Pingliang red cattle exhibit a closer genetic affinity to Chinese cattle compared to common *B. taurus* and *B. indicus* breeds from Europe. Previous studies have established a correlation between genetic distances among populations and their geographic distribution, indicating that populations in close proximity are more likely to share a common ancestor [38]. Our research reveals a close relationship between Qinchuan and Chinese cattle, with Pingliang red cattle exhibiting the largest shared IBD fragment with it. This can be attributed to their relatively close geographical proximity as well as Qinchuan’s ancestral role in shaping the genetics of Pingliang red cattle.

## 5. Conclusions

The present study offers the initial comprehensive analysis of the genetic structure of Pingliang red cattle using genome-wide SNP markers. By employing PCA, admixture, phylogenetic tree, identical by descent, and TreeMix analyses, it was revealed that Pingliang red cattle exhibit distinct genetic characteristics that clearly differentiate them from other breeds. Furthermore, they can be classified as a hybrid lineage originating from *B. taurus* and *B. indicus*.

## Data Availability

Publicly available datasets were analyzed in this study. These data can be found as follows: WIDDE (http://widde.toulouse.inra.fr/widde/widde/main.do;jsessionid=114993EC02C5CB17D1FA786DD4F2337A?module=cattle# (accessed on 12 May 2022)), NCBI GEO (https://www.ncbi.nlm.nih.gov/geo/ (accessed on 12 May 2022)).
